# Thermoresponsive Gels with Rosemary Essential Oil: A Novel Topical Carrier for Antimicrobial Therapy and Drug Delivery Applications

**DOI:** 10.3390/gels11010061

**Published:** 2025-01-12

**Authors:** Ludovic Everard Bejenaru, Adina-Elena Segneanu, Cornelia Bejenaru, Ionela Amalia Bradu, Titus Vlase, Dumitru-Daniel Herea, Marius Ciprian Văruţ, Roxana Maria Bălăşoiu, Andrei Biţă, Antonia Radu, George Dan Mogoşanu, Maria Viorica Ciocîlteu

**Affiliations:** 1 Department of Pharmacognosy & Phytotherapy, Faculty of Pharmacy, University of Medicine and Pharmacy of Craiova, 2 Petru Rareş Street, 200349 Craiova, Romania; ludovic.bejenaru@umfcv.ro (L.E.B.); andreibita@gmail.com (A.B.); george.mogosanu@umfcv.ro (G.D.M.); 2 Institute for Advanced Environmental Research, West University of Timişoara, 4 Oituz Street, 300086 Timişoara, Romania; adina.segneanu@e-uvt.ro (A.-E.S.); ionela.bradu@e-uvt.ro (I.A.B.); titus.vlase@e-uvt.ro (T.V.); 3 Department of Pharmaceutical Botany, Faculty of Pharmacy, University of Medicine and Pharmacy of Craiova, 2 Petru Rareş Street, 200349 Craiova, Romania; antonia.radu@umfcv.ro; 4 Research Center for Thermal Analyzes in Environmental Problems, West University of Timişoara, 16 Johann Heinrich Pestalozzi Street, 300115 Timişoara, Romania; 5 National Institute of Research and Development for Technical Physics, 47 Dimitrie Mangeron Avenue, 700050 Iaşi, Romania; dherea@phys-iasi.ro; 6 Department of Physics, Faculty of Pharmacy, University of Medicine and Pharmacy of Craiova, 2 Petru Rareş Street, 200349 Craiova, Romania; marius.varut@umfcv.ro; 7 Department of Biochemistry, Faculty of Pharmacy, University of Medicine and Pharmacy of Craiova, 2 Petru Rareş Street, 200349 Craiova, Romania; roxana.balasoiu@umfcv.ro; 8 Department of Instrumental and Analytical Chemistry, Faculty of Pharmacy, University of Medicine and Pharmacy of Craiova, 2 Petru Rareş Street, 200349 Craiova, Romania; maria.ciocilteu@umfcv.ro

**Keywords:** rosemary essential oil, PLGA, nanoformulation, thermoresponsive gel, wound healing, antimicrobial, topical drug delivery

## Abstract

This study investigates the development and comprehensive characterization of innovative thermoresponsive gels incorporating rosemary essential oil (RoEO) encapsulated in poly(lactic-*co*-glycolic acid) (PLGA) microparticles, with a focus on their potential applications in topical antimicrobial and wound healing therapies. RoEO, renowned for its robust antimicrobial, antioxidant, and wound-healing properties, was subjected to detailed chemical profiling using gas chromatography-mass spectrometry (GC–MS), which identified oxygenated monoterpenes as its dominant constituents. PLGA microparticles were synthesized through an optimized oil-in-water emulsion technique, ensuring high encapsulation efficiency and structural integrity. These microparticles were thoroughly characterized using Fourier-transform infrared (FTIR) spectroscopy to confirm functional group interactions, scanning electron microscopy (SEM) for surface morphology, X-ray diffraction (XRD) for crystalline properties, and thermal analysis for stability assessment. The synthesized microparticles displayed uniform size distribution and efficient encapsulation, demonstrating compatibility with the gel matrix. Two distinct thermoresponsive gel formulations were developed using varying ratios of Poloxamer 407 and Poloxamer 188 to achieve optimal performance. The gels were evaluated for key physicochemical properties, including pH, gelation temperature, viscosity, and rheological behavior. Both formulations exhibited thermoresponsive gelation at skin-compatible temperatures (27.6 °C and 32.9 °C), favorable pH levels (6.63 and 6.40), and shear-thinning behavior suitable for topical application. Antimicrobial efficacy was assessed against common pathogens associated with skin infections, including *Staphylococcus aureus*, *Escherichia coli*, and *Candida albicans*. The RoEO-PLGA-loaded gels demonstrated significant inhibitory effects, confirming their potential as effective carriers for controlled and localized drug delivery. These findings underscore the promising application of RoEO-PLGA-loaded thermoresponsive gels in addressing challenges associated with topical antimicrobial therapies and wound care, offering an innovative approach to enhancing therapeutic outcomes. By integrating the bioactive potential of RoEO with the advanced delivery capabilities of PLGA microparticles and thermoresponsive gels, this study paves the way for the development of next-generation formulations tailored to meet the specific needs of localized drug delivery in skin health management.

## 1. Introduction

Thermoresponsive gels, a fascinating class of smart materials, have gained significant attention in recent years due to their ability to undergo reversible changes in physical properties in response to temperature variation [1]. These gels are typically composed of polymer networks that are sensitive to temperature, leading to unique behaviors such as sol-gel transitions or changes in volume and mechanical properties [2]. This responsiveness opens up a multitude of applications across diverse fields, ranging from biomedicine [3] to environmental engineering [4]. The distinctive characteristic of some thermoresponsive gels lies in their critical temperature thresholds, lower critical solution temperature (LCST), and upper critical solution temperature. Below or above these temperatures, the gel undergoes a phase transition. For instance, polymers such as poly(N-isopropylacrylamide) (PNIPAAm) exhibit an LCST around 32 °C, making them ideal for applications that require a response near physiological conditions [5]. The molecular mechanisms behind this transition involve changes in hydrophilic and hydrophobic interactions, driven by alterations in hydrogen bonding and polymer-solvent affinities [6]. For poloxamers, the transition is not strictly an LCST phase separation but rather a shift in the self-assembly behavior, where the transition of the polymer from being solubilized in water to forming micelles and subsequently a gel network [7]. Poloxamers (also known as Pluronics) are triblock copolymers composed of poly(ethylene oxide) (PEO) and poly(propylene oxide) (PPO) in a PEO–PPO–PEO configuration [8]. Their thermoresponsiveness is attributed to the temperature-dependent micellization and gelation of the polymers in aqueous solutions. Poloxamer 407 typically forms a gel when heated to temperatures around 30–37 °C depending on concentration [9]. This temperature range makes Poloxamer 407 suitable for biomedical applications such as topical or injectable drug delivery systems [10]. Poloxamer 188 has a lower hydrophobic PPO content than Poloxamer 407, leading to weaker thermoresponsive behavior.

Thermoresponsive gels are advantageous because they exhibit a sol-to-gel transition at specific temperatures, often around physiological skin temperatures (33–37 °C). This property enables the gel to remain liquid at room temperature (RT), facilitating easy application, gelate upon skin contact, and ensuring prolonged retention at the site of application, minimizing runoff or displacement.

Thermoresponsive gels have revolutionized the biomedical field by enabling innovations in drug delivery [11], tissue engineering, and wound healing [12]. In drug delivery systems, these gels can act as carriers that release therapeutic agents in a controlled manner when the temperature reaches a specific threshold. For example, thermoresponsive hydrogels can be injected in a liquid state and solidified at body temperature, ensuring localized and sustained drug delivery [13]. In tissue engineering, thermoresponsive scaffolds provide a conducive environment for cell growth and tissue regeneration. By adjusting the temperature, these gels can facilitate the encapsulation and subsequent release of cells or bioactive molecules, making them invaluable tools for regenerative medicine.

Beyond healthcare, thermoresponsive gels offer solutions to pressing environmental challenges. They are employed in wastewater treatment for selective adsorption and removal of pollutants. The temperature-dependent swelling and shrinking properties of these gels allow for efficient capture and release cycles, making them energy-efficient and reusable.

In industrial settings, thermoresponsive gels find utility in smart coatings, sensors, and actuators. Their ability to modulate properties such as adhesion, transparency, and conductivity with temperature changes makes them integral to advanced manufacturing and robotics.

*Rosmarinus officinalis* is an evergreen, perennial shrub of the *Lamiaceae* family [14]. Although it originates in the Mediterranean region, it is nowadays cultivated worldwide, due to both its economic importance and richness in bioactive compounds [15]. Famous in gastronomy thanks to its aromatic evergreen leaves that possess a unique flavor, rosemary has also been evaluated by the European Food Safety Authority (EFSA) and it is safely used as a natural preservative as it prevents oxidation processes and microbial contamination [16]. Rosemary was used in traditional medicine as a spasmolytic, to alleviate muscle spasms, renal colic, and dysmenorrhea, to treat respiratory diseases, enhance intellectual performance, and promote hair growth [17].

Recent research highlights *R. officinalis* as a multifaceted medicinal plant with a wide range of pharmacological activities. Notably, it demonstrates potent antioxidant [18], antiproliferative, and cytotoxic effects in vitro [19,20,21], making it a candidate for cancer therapy. Its anti-inflammatory properties [22] contribute to its potential in managing chronic inflammatory conditions. Additionally, *R. officinalis* exhibits antiviral activity [23], which could be leveraged in the development of antiviral therapeutics. Importantly, its anticholinesterase activity [18] and neuroprotective effects show promise in addressing nervous system disorders, including anxiety, depression, epilepsy, and Alzheimer’s disease [24], underscoring its potential as a therapeutic adjuvant in neurodegenerative and psychiatric disorders.

The phytochemical composition of rosemary essential oil (RoEO) has been extensively studied, revealing 150 different compounds, with 1,8-cineole, camphor and α-pinene being major compounds [25]. These biologically active compounds prompt interest among researchers of natural antioxidants, antimicrobial and anti-tumoral agents [15,26,27].

This study aims to develop and characterize advanced thermoresponsive gels incorporating RoEO encapsulated within poly(lactic-*co*-glycolic acid) PLGA nanoparticles (NPs), offering a groundbreaking approach to topical antimicrobial therapy. This research introduces a novel synergy between the potent bioactive properties of RoEO and the precision-controlled delivery afforded by PLGA NPs, embedded within a smart gel matrix for enhanced functionality.

Key objectives include: (i) comprehensive chemical profiling of RoEO using cutting-edge analytical techniques to elucidate its therapeutic potential; (ii) the innovative design and optimization of PLGA NPs with high encapsulation efficiency, stability, and sustained release profiles; and (iii) the formulation of thermoresponsive gels with highly tunable physicochemical properties, such as pH, gelation temperature, and rheological behavior, to ensure efficient application and user comfort.

The study will also assess the gels’ antimicrobial performance against multidrug-resistant pathogens, along with their wound healing efficacy and suitability for skin care applications. By addressing critical challenges in localized drug delivery systems—such as poor stability, inadequate release control, and limited efficacy against resistant strains—this work pioneers an integrated platform that bridges natural product innovation with advanced material science. This multifaceted approach offers significant potential to redefine therapeutic strategies for topical infections and skin care.

The research employs comprehensive characterization methods, including gas chromatography-mass spectrometry (GC–MS) for the detailed chemical profiling of RoEO, as well as Fourier-transform infrared (FTIR) spectroscopy, scanning electron microscopy (SEM), X-ray powder diffraction (XRD), thermal analysis, dynamic light scattering (DLS) to analyze the physical properties of the microparticles, and antibacterial/antifungal activity. The study also highlights the unique thermoresponsive properties of the developed gels, which exhibit shear-thinning and thixotropic behavior. This work aims to explore the feasibility of these formulations for localized drug delivery, addressing current gaps in therapeutic options for topical infections and skin care.

## 2. Results and Discussion

### 2.1. GC–MS Analysis

**Figure 1:**
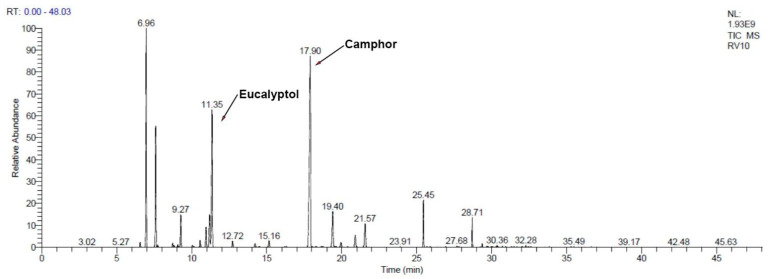
GC–MS chromatogram of RoEO Tunisia reference. GC: Gas chromatography; MS: Mass spectrometry; RoEO: Rosemary essential oil.

**Figure 2:**
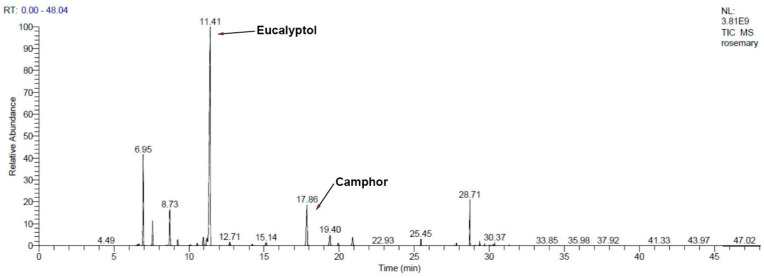
GC–MS chromatogram of RoEO sample.

GC–MS analysis revealed that monoterpenes (both hydrocarbons and oxygenated) dominate the composition (Table 1; Figure 1 and Figure 2), contributing to the antibacterial potential of RoEO. The high percentage of oxygenated monoterpenes (58.7% in RoEO and 68.46% in the RoEO Tunisia reference) enhances the overall antimicrobial efficacy.

### 2.2. FTIR Analysis

**Figure 3:**
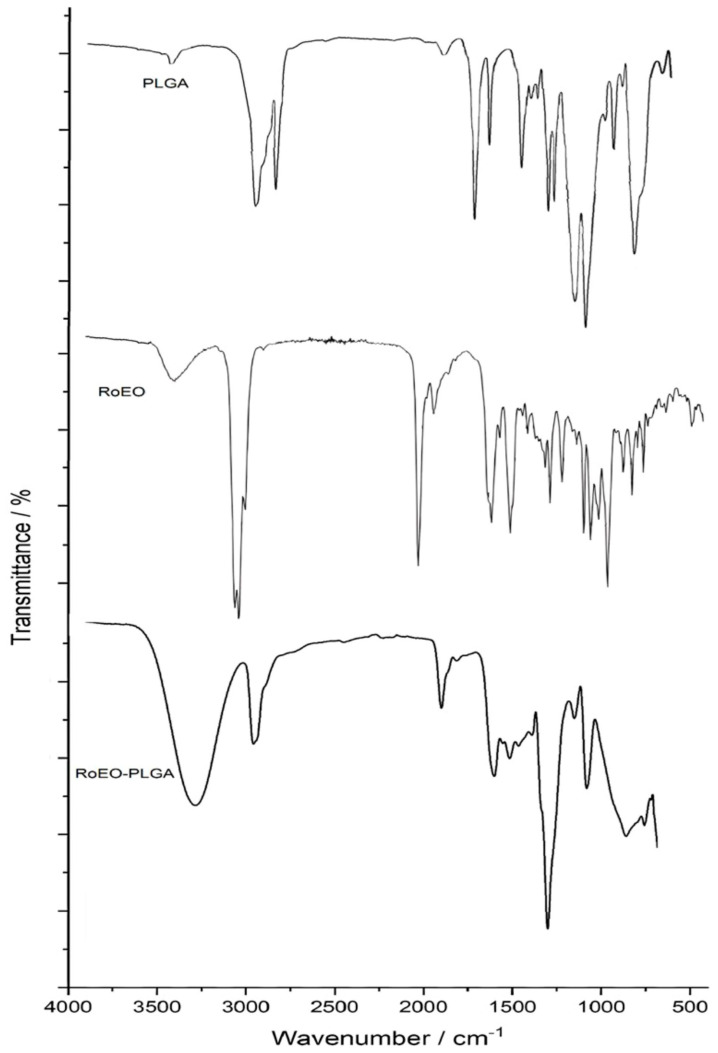
FTIR spectra for PLGA, RoEO, and RoEO-PLGA samples. FTIR: Fourier-transform infrared; PLGA: Poly(lactic-*co*-glycolic) acid; RoEO: Rosemary essential oil.

The FTIR spectrum of PLGA (Figure 3) exhibits its characteristic adsorption peaks, providing clear evidence of its molecular structure [28,29]. The peak at approximately 3012 cm^−1^ corresponds to the –CH(CH_3_)– groups, indicative of the polymer’s aliphatic nature [28,29]. The strong adsorption band at 1754 cm^−1^ is attributed to C=O stretching vibrations, a hallmark of the carbonyl groups in the ester linkages of PLGA [28,29]. Additionally, peaks at 1452 cm^−1^, 1180 cm^−1^, and 1133 cm^−1^ correspond to the carbonyl C–O bond, the C–O–C ether group, and the C–H vibrations of methyl groups, respectively [28].

The FTIR spectrum of RoEO (Figure 3) highlights its chemically diverse composition, primarily consisting of monoterpenes, oxygenated monoterpenes, sesquiterpenes, and related compounds. The broad peak at approximately 3434 cm^−1^ corresponds to O–H stretching vibrations, indicating the presence of hydroxyl groups likely associated with oxygenated monoterpenes or sesquiterpenes [30]. The peak at ~2932 cm^−1^ reflects C–H stretching vibrations, characteristic of aliphatic hydrocarbons [30]. A strong peak at ~1746 cm^−1^ corresponds to C=O stretching vibrations, attributed to carbonyl groups in esters, aldehydes, or ketones [30]. The peak at ~1088 cm^−1^ represents C–O stretching vibrations, characteristic of ether or alcohol groups [30]. Additionally, the peak at ~812 cm^−1^ corresponds to out-of-plane bending vibrations of C–H bonds, indicative of aromatic or alkyl groups, while peaks between ~1300–1500 cm^−1^ represent C–H bending and C–O–C vibrations associated with ester groups [30].

The FTIR spectrum of the RoEO-PLGA formulation (Figure 3) confirms the successful encapsulation of RoEO within the PLGA matrix. A broad band at 3305 cm^−1^ corresponds to O–H stretching vibrations, signifying the presence of hydroxyl groups, likely contributed by both RoEO and PLGA [28,29]. The peak at ~2932 cm^−1^ reflects C–H stretching vibrations from aliphatic hydrocarbons, characteristic of both the EO and the polymer [27,28,29]. A prominent peak at ~1758 cm^−1^, representing C=O stretching vibrations, is a defining feature of the ester groups in PLGA [28,29]. Peaks at ~1423 cm^−1^ and ~1328 cm^−1^ correspond to C–H bending and C–O–C vibrations, respectively. Additional peaks at ~1270 cm^−1^, ~1189 cm^−1^, and ~1084 cm^−1^ reflect C–O stretching vibrations, while peaks at ~917 cm^−1^ and ~840 cm^−1^ correspond to out-of-plane C–H bending vibrations, indicative of alkyl groups. In the fingerprint region, peaks at 602 cm^−1^ and 478 cm^−1^ reflect deformation vibrations unique to PLGA [28,29]. Overlapping peaks, likely due to structural similarities between PLGA and poly(vinyl alcohol) (PVA), suggest intricate molecular interactions within the encapsulation system [28,29]. These comprehensive spectral findings validate the integration of RoEO into the PLGA system, demonstrating the effectiveness and efficiency of the encapsulation process.

### 2.3. XRD Analysis

**Figure 4:**
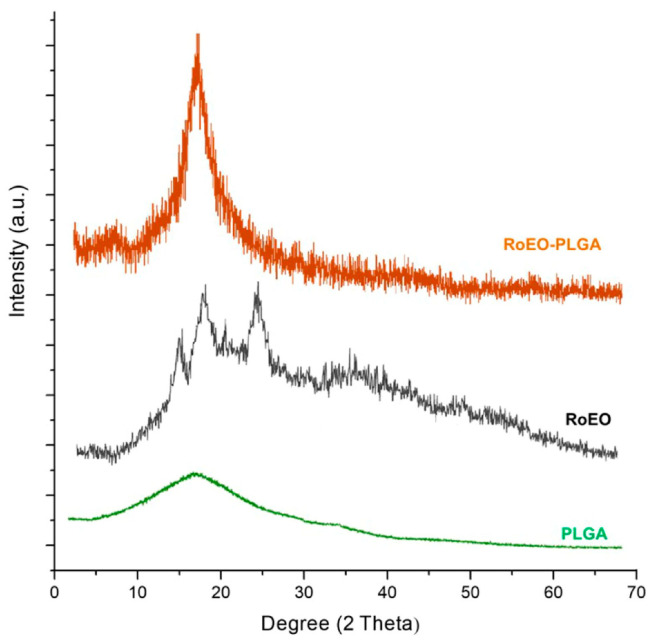
XRD pattern of PLGA, RoEO, and RoEO-PLGA samples. XRD: X-ray diffraction.

XRD analysis of the RoEO-PLGA sample (Figure 4) reveals a characteristic diffraction pattern that is predominantly amorphous in nature. The broad diffraction peak centered around 2-theta (2*θ*°) indicates the absence of significant crystalline regions, which is typical of polymeric materials like PLGA [29,31]. This broad peak suggests a highly disordered structure within the polymer matrix, consistent with its amorphous nature [29,31].

The XRD analysis of the RoEO sample (Figure 4) demonstrates a broad diffraction peak in the 15–23° 2*θ* region, characteristic of organic compounds with semi-crystalline or amorphous properties. This broad peak reflects the intrinsic structural complexity of RoEO, which is composed of diverse bioactive molecules [32].

In contrast, the PLGA sample (Figure 4) exhibits a fully amorphous profile with no distinct diffraction peaks, consistent with the disordered molecular arrangement typical of polymeric materials [29].

In the RoEO-PLGA formulation, the absence of sharp, well-defined peaks in the diffraction pattern further confirms the successful encapsulation of RoEO into the PLGA matrix. The incorporation of RoEO does not induce any degree of crystallinity but rather preserves or even enhances the amorphous nature of the system. This structural feature is particularly advantageous for drug delivery applications, as amorphous systems are often associated with improved solubility, enhanced bioavailability, and controlled release profiles of active components.

The observed XRD pattern highlights the excellent structural compatibility between RoEO and PLGA, ensuring a stable and homogeneous encapsulated system. This compatibility, coupled with the amorphous nature of the formulation, reinforces its potential as a robust platform for advanced therapeutic applications, offering improved performance and reliability in drug delivery systems.

### 2.4. Morphological Studies

#### 2.4.1. SEM Analysis

**Figure 5:**
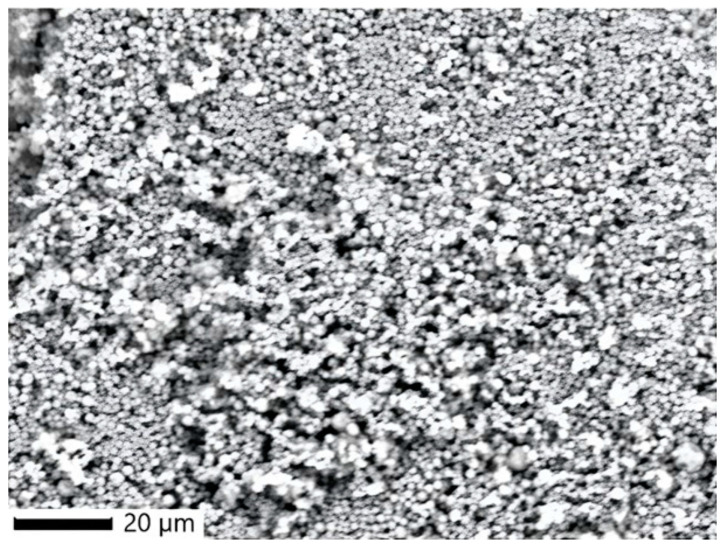
Morphological aspects of RoEO-PLGA microparticles (SEM image). SEM: Scanning electron microscopy.

SEM image of RoEO-PLGA microparticles (Figure 5) synthesized using the emulsion method shows clear, individual particle boundaries, indicating that the emulsion used during synthesis was stable, preventing significant coalescence or irregularities during NP formation. The particles appear roughly spherical, which is a common feature of particles synthesized via the emulsion method [33]. The smoothness and uniformity of the particles suggest a well-controlled formulation process. The particles exhibit a polydisperse size distribution, but further analysis (DLS) provided a clearer understanding. Some degree of particle aggregation is visible, likely due to the drying process before SEM imaging.

#### 2.4.2. EDS Spectrum

**Figure 6:**
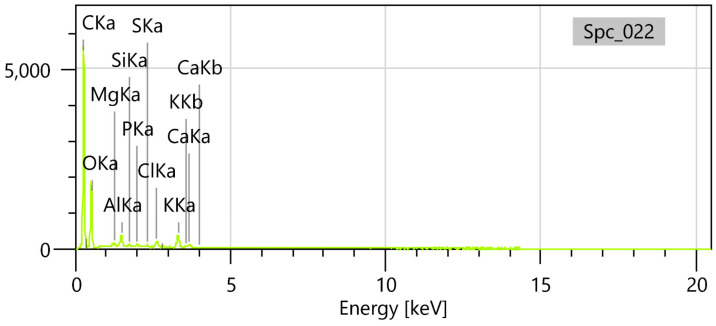
EDS spectrum of RoEO-PLGA microparticles. EDS: Energy-dispersive X-ray spectroscopy.

The EDS spectrum highlights prominent peaks for carbon and oxygen, which are primarily attributed to the polymer backbone of PLGA (Figure 6), composed of lactic and glycolic acid units [29]. These elements are also contributed by the RoEO sample, reflecting its organic composition. Notably, the presence of magnesium (Mg), calcium (Ca), and potassium (K), along with silicon (Si), aluminum (Al), and trace amounts of sulfur (S) and phosphorus (P), is likely derived from the RoEO, consistent with the natural mineral and elemental profile of RoEOs [34].

The detection of chlorine, however, can be attributed to the use of PVA as a surfactant during the emulsion process. This element’s presence, absent in pure RoEO, signifies a hallmark of the encapsulation process, further distinguishing the RoEO-PLGA system. Together, these findings provide a detailed and precise characterization of the elemental composition, offering insights into both the intrinsic properties of the RoEO and the structural integration within the PLGA matrix.

#### 2.4.3. DLS Analysis

**Figure 7:**
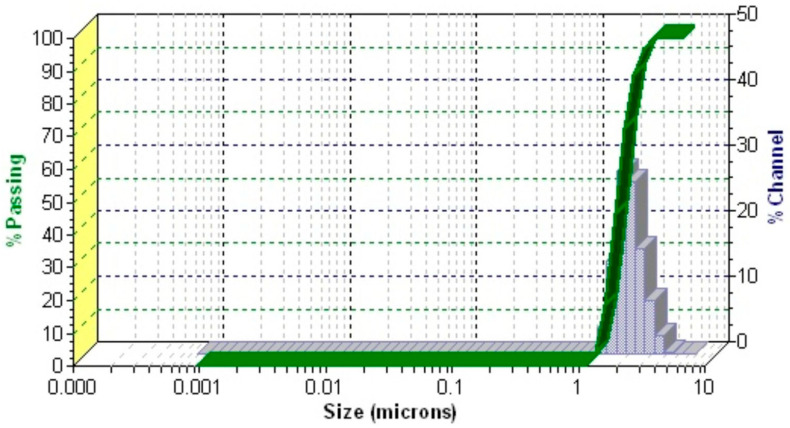
DLS pattern of RoEO-PLGA sample.

DLS analysis, a precise, sensitive, and non-destructive technique, is widely utilized for evaluating particle size and size distribution in colloidal systems [30]. The DLS profile of the RoEO-PLGA system (Figure 7) exhibits a single, well-defined peak, indicating a monodisperse particle population.

The analysis revealed a mean particle diameter of 1.98 μm and a polydispersity index (PDI) of 0.061, underscoring the highly uniform particle size distribution of the system.

### 2.5. Thermal Analysis

Thermal analysis was conducted to assess the thermal stability and decomposition profiles of the formulations. This was important for confirming that the encapsulated RoEO and the PLGA matrix remain stable under storage and application conditions.

**Figure 8:**
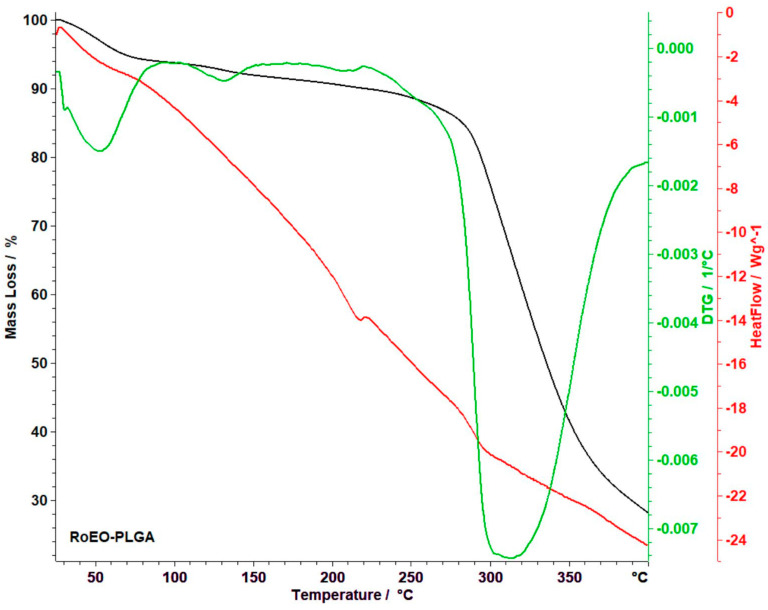
Thermoanalytical data for RoEO-PLGA sample (black line: TG analysis; green line: DTG analysis; red line: HF). DTG: Derivative thermogravimetry; HF: Heat flow; TG: Thermogravimetry.

The thermal behavior of the RoEO-PLGA sample, as depicted in Figure 8, integrates data from thermogravimetric analysis (TG, black line), derivative thermogravimetry (DTG, green line), and heat flow (HF, red line) over the temperature range of 30–400 °C, offering a comprehensive understanding of its decomposition process.

The analysis reveals a total mass loss of 69.82%, highlighting distinct thermal events and transitions.

The thermal decomposition of the RoEO-PLGA system is revealed through DTG curves, which identify distinct stages of degradation with remarkable precision. The first stage, occurring below 85 °C, is associated with the evaporation of water, accounting for a 5.40% mass loss. The DTG curve displays a sharp peak at 73 °C, indicating the maximum moisture removal rate. This sharp peak highlights a rapid evaporation process, underscoring the efficient removal of water or residual solvents, which is crucial for ensuring the stability of the formulation in moisture-sensitive applications. The second stage, between 105 °C and 164 °C, corresponds to the initial thermal degradation of the PLGA matrix and potentially the encapsulated RoEO. This stage marks the onset of structural breakdown, as the DTG curve reveals an increase in the rate of mass loss, transitioning from moisture evaporation to the early stages of polymer degradation. The DTG peak in this range precisely indicates the temperature at which the material starts to lose its structural integrity, signaling the breakdown of both the polymer and volatile components. Understanding this stage is critical for ensuring the thermal stability of the system and for designing processes that avoid premature degradation.

A third stage, occurring between 187 °C and 223 °C, accounts for a minor mass loss of 1.10% and reflects the decomposition of smaller or residual volatile components. While this stage is less prominent, the DTG curve accurately identifies the temperature at which these volatiles are released, offering clarity on the less significant degradation processes. This differentiation between major and minor events enhances the understanding of the system’s behavior, isolating critical thermal transitions from secondary effects.

The fourth stage, which spans 238 °C to 393 °C, is the most significant, marked by a substantial 59.47% mass loss. The DTG peak at 314 °C corresponds to the cleavage of ester bonds in the PLGA polymer, marking the main thermal degradation phase. This is the most critical decomposition step, as it signifies the breakdown of the polymer backbone and the potential release of the encapsulated RoEO. The significant mass loss during this stage highlights the polymer’s vulnerability to thermal stress, emphasizing the need for carefully managed thermal conditions to preserve the system’s integrity, particularly in applications such as controlled release and material stability.

These stages outlined by the DTG curve provide an in-depth understanding of the RoEO-PLGA system’s thermal behavior, offering key insights into its stability and decomposition processes.

In addition to DTG analysis, the HF curve provides further insight into the thermal transitions of the sample. An endothermic peak below 100 °C corresponds to the evaporation of residual moisture or solvents, while a pronounced exothermic peak between 200 °C and 300 °C indicates the oxidative decomposition of both PLGA and RoEO components, signaling crucial structural and chemical changes.

This comprehensive thermal analysis, integrating data from TG, DTG, and HF curves, underscores the complex and dynamic behavior of the RoEO-PLGA system under thermal stress. The distinct thermal events observed not only confirm the stability of the encapsulated system but also offer invaluable insights into its performance across various conditions. These findings validate the RoEO-PLGA system’s potential for applications requiring precise thermal control, such as controlled drug delivery, wound healing, and advanced material formulations.

### 2.6. Gelation Temperature and pH

Gelation temperature is a crucial factor in developing an in situ gelling thermosensitive gel. The optimized formulation must remain in liquid form at RT, while rapidly transitioning to a gel state upon administration on the skin, which is influenced by the physiological temperature of the skin. The average skin temperature for healthy adults typically ranges from 33 °C to 37 °C (approximately 92.3° F to 98.4° F) [35]. This temperature can vary based on several factors, including body region, with certain areas like the forehead and neck generally exhibiting higher temperatures compared to the palms or forearms [36]. Additionally, skin temperature can fluctuate due to environmental conditions and physical activity, reflecting the body’s thermoregulation processes. Therefore, this study was deliberately conducted as an initial assessment to identify the appropriate concentration of P407 for the formulation, based on the target gelation temperature and time.

The investigation was conducted using two different concentrations of Poloxamer 407 and Poloxamer 188 (20% and 25% *w*/*v*, respectively 2% and 10%, *w*/*v*).

The pH of RoEO-PLGA _A and RoEO-PLGA _B samples was also investigated. The optimal pH for a gel intended for application on the skin is typically between 5 and 6.5. This range is considered slightly acidic, which aligns with the natural pH of healthy skin, usually around 5 [37]. Maintaining this pH level is crucial as it helps support the skin’s barrier function, prevents irritation, and promotes overall skin health. Using products within this optimal pH range can enhance their effectiveness and minimize the risk of adverse reactions such as dryness or irritation [38] and also can significantly affect the permeation of active ingredients through the skin barrier. A slight increase was observed for the formulation containing 25% (*w*/*v*) Poloxamer 407, compared with the formulation containing 20% (*w*/*v*) Poloxamer 407 (Table 2).

### 2.7. Viscosity and Rheological Behavior

RoEO-PLGA _A and RoEO-PLGA _B gel formulations were tested for the behavior of the gel during increasing shear rate (ramp-up, blue line) and decreasing shear rate (ramp-down, red line). This type of test was performed to assess whether the two formulations exhibit thixotropy or hysteresis in their flow behavior (Table 3).

The thixotropic index (TI) was calculated as the ratio of two viscosities corresponding to low rotational speeds (1:10 ratio). The supra-unitary TI values confirm that RoEO-PLGA_B is a shear-thinning, non-Newtonian liquid. Additionally, the large nonzero yield stress value for RoEO-PLGA_B reflects its plastic rheology. Conversely, the small yield stress value for RoEO-PLGA_A also indicates a plastic rheological behavior.

The energy dissipation ratio (EDR) was calculated as the ratio of the hysteresis area to the area under the ramp-up curve. The negative EDR value for RoEO-PLGA_B is a direct consequence of its negative hysteresis area. Interestingly, the magnitude of the EDR is higher for RoEO-PLGA_A despite its smaller hysteresis area, as the area under the ramp-up curve is significantly lower, consistent with the properties of a low-viscosity liquid.

The shear recovery of the liquids was evaluated at a shear rate of 12.5 s^−1^. This pa-rameter was calculated as the ratio of viscosities, at the specified shear rate, associated with the ramp-up and ramp-down curves, respectively.

**Figure 9:**
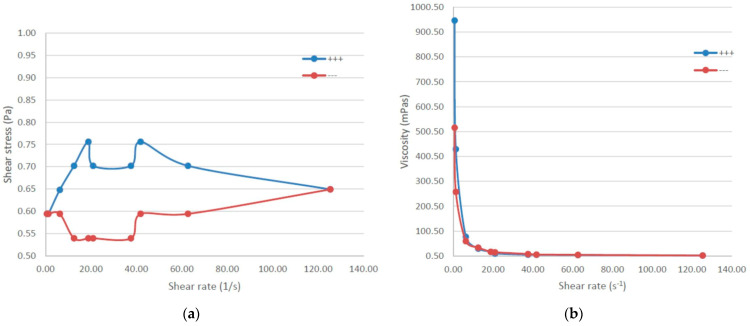
Rheological behavior of RoEO-PLGA _A: (**a**) Relationship between shear stress and shear rate (blue—increasing shear rate; red—decreasing shear rate); (**b**) Viscosity under varying shear rates (at 36 °C; blue—increasing shear rate; red—decreasing shear rate).

For RoEO-PLGA _A formulation shear stress initially increases with shear rate (Figure 9a, blue series), reaching a peak around 20 s^−1^, and shows a slight plateau or oscillation between 20–40 s^−1^. It gradually decreases as the shear rate continues to rise. When decreasing the shear rate (Figure 9a, red series), the shear stress shows less variability and a more consistent trend. The shear stress gradually increases as the shear rate decreases, converging with the blue series near 140 s^−1^.

The discrepancy between the two series indicates thixotropy (Figure 9a), a time-dependent shear thinning property. During ramp-up (blue series), the gel resists deformation more strongly, as indicated by higher shear stress values. During ramp-down (red series), the gel shows lower resistance to shear, reflecting structural breakdown under shear stress.

RoEO-PLGA _A viscosity is consistent across both increasing and decreasing shear rates (Figure 9b), implying that it has a stable rheological response under these conditions. At very low shear rates, the viscosity is high, reaching above 25,000 mPa·s for both series. As the shear rate increases, the viscosity rapidly decreases and stabilizes at a much lower value beyond a shear rate of approximately 40 s^−1^. The close overlap indicates that the gel quickly returns to its original structure or is not significantly affected by the applied shear.

**Figure 10:**
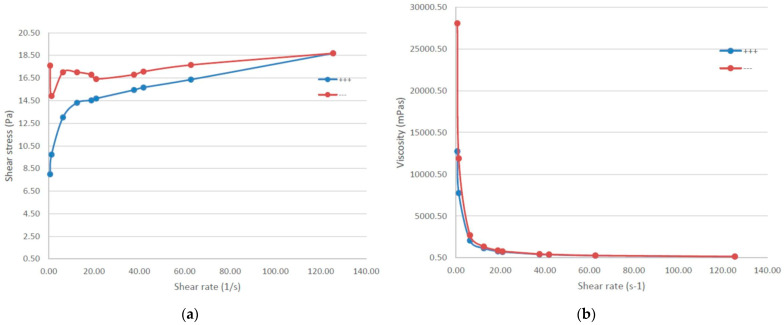
Rheological behavior of RoEO-PLGA _B: (**a**) Relationship between shear stress and shear rate (blue—increasing shear rate; red—decreasing shear rate); (**b**) Viscosity under varying shear rates (at 36 °C; blue—increasing shear rate; red—decreasing shear rate).

RoEO-PLGA _B exhibits shear-thinning properties, where shear stress does not increase linearly with shear rate. This is consistent with Poloxamer gels, which align and reorganize under shear, reducing internal resistance (Figure 10a). The mismatch between the blue and red curves at lower shear rates suggests thixotropy, where the gel structure breaks down under shear but does not immediately recover. The hysteresis loop (difference between blue and red series) indicates time-dependent structural rearrangements in the gel. This is more pronounced at lower shear rates (Figure 10b).

The rheological behavior of RoEO-PLGA_B exhibits two noteworthy characteristics, as illustrated in the graphical representation in Figure 10: (a) the shear stress curve corresponding to the ramp-up curve (blue) lies below the ramp-down curve (red), and (b) the viscosity decreases with increasing shear rate, yet the red curve (decreasing shear rate) remains above the blue curve (increasing shear rate). The observed decline in viscosity with increasing shear rate confirms that RoEO-PLGA_B is a non-Newtonian plastic liquid.

These particularities suggest that the shear stress increases following the application of shear, indicating that the internal structure of the liquid becomes more organized. Furthermore, the viscosity of RoEO-PLGA_B is higher after being subjected to shear for a certain period (at varying shear rates). This distinctive behavior strongly suggests that RoEO-PLGA_B exhibits rheopectic properties, a phenomenon rarely observed in conjunction with plastic rheology.

In contrast, thixotropic liquids, such as RoEO-PLGA_A, typically exhibit behavior characterized by lower shear stress after the material is sheared (the blue curve above the red curve). Similarly, their viscosity decreases following shear exposure, with the viscosity curve for the increasing shear rate (blue) positioned above that of the decreasing shear rate (red), even in cases where the thixotropic area is small.

The hysteresis area was determined as the difference between the area under the ramp-up curve and the area under the ramp-down curve. The negative hysteresis area observed for RoEO-PLGA_B indicates a structural build-up liquid as a consequence of the increased shear rate.

### 2.8. Antimicrobial Activity

Antimicrobial screening tests of RoEO, RoEO Tunisia reference, RoEO-PLGA _A, and RoEO-PLGA _B were performed in vitro using agar well diffusion method against one Gram-positive strain (*Staphylococcus aureus*), one Gram-negative strain (*Escherichia coli*) and a fungal strain (*Candida albicans*). Results are shown in Table 4. The inhibition zones (IZs) ranged from 9.9 to 27.2 mm against all tested microorganisms.

**Figure 11:**
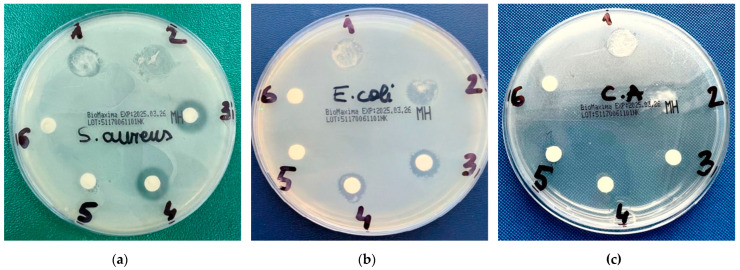
Antimicrobial screening against (**a**) *S. aureus*, (**b**) *E. coli*, and (**c**) a fungal strain (*C. albicans*).

The most significant inhibitory activity was observed against *C. albicans*, highlighting its enhanced susceptibility to the formulations. Interestingly, the RoEO sample exhibited slightly larger IZs than the RoEO Tunisia reference, emphasizing potential differences in composition or activity (Figure 11a–c).

The antimicrobial activity measured by the IZs depends on the diffusion rates of the active compounds through the agar medium. The encapsulated RoEO in PLGA microparticles may exhibit slower diffusion compared to free RoEO due to the controlled release mechanism, which could explain the slightly smaller IZs for *S. aureus* and *E. coli* in the RoEO-PLGA gels compared to pure RoEO.

In contrast, the larger IZs for *C. albicans* with RoEO-PLGA gels (27.0–27.2 mm) compared to pure RoEO (10.8–12.8 mm) suggest that the sustained release of bioactive compounds maintains an effective concentration over a broader area, enhancing antifungal efficacy.

The concentration of active compounds at the edge of the diffusion zone can vary depending on the release kinetics of the formulation. For *C. albicans*, the larger IZs observed with RoEO-PLGA gels (27.0–27.2 mm) compared to pure RoEO (10.8–12.8 mm) likely result from the sustained release of bioactive compounds, maintaining an effective concentration over a larger area.

The encapsulation of RoEO within PLGA microparticles facilitates a controlled release profile, which likely contributes to the enhanced antifungal activity against *C. albicans*. This controlled release mechanism may be less significant for bacterial strains like *S. aureus* and *E. coli,* where the immediate availability of the active compounds is more critical.

The observed differences may also arise from variations in the susceptibility of micro-bial cell walls to the active compounds. The thicker cell wall of Gram-positive *S. aureus* and the outer membrane of Gram-negative *E. coli* may influence the diffusion and efficacy of the antimicrobial agents differently compared to fungal cells.

### 2.9. Discussion

Recent advancements in hydrogel-based therapies have demonstrated significant potential in treating complex wounds, such as pressure ulcers and diabetic foot ulcers (DFUs). For instance, Qi et al. developed an adenosine triphosphate (ATP)-activated, spatiotemporally controlled hydrogel prodrug system that effectively treats multidrug-resistant bacteria-infected pressure ulcers by generating reactive oxygen species (ROS) in response to bacterial ATP, thereby enhancing antibacterial efficacy [39]. Similarly, another study introduced an immunomodulatory hydrogel capable of self-cascade glucose depletion and ROS scavenging, addressing hyperglycemia-induced inflammation and oxidative stress in DFU wounds, leading to improved healing outcomes [40].

These innovative hydrogel systems exemplify the integration of responsive and multifunctional properties tailored to the specific pathophysiological conditions of chronic wounds, offering promising avenues for advanced wound care management.

In recent years, poloxamers have found extensive applications in various fields, including drug formulation [11,41], prevention of tissue adhesion, and wound care [42]. Poloxamers provide a bio-inert environment [43] due to the hydrophilicity and flexibility of the PEO chains. As a result, most cells do not proliferate on these polymers, which have been utilized as barriers to tissue adhesion.

Poloxamer 407 and Poloxamer 188 are known for their thermo-reversible gelation and shear-thinning behavior. Poloxamer 407 and Poloxamer 188 are amphiphilic triblock copolymers with hydrophilic PEO and hydrophobic PPO segments. Their thermoresponsiveness arises from temperature-dependent micellization and gelation. At low temperatures, hydrogen bonding between water molecules and the hydrophilic PEO segments prevents micelle formation, resulting in a sol-like state. As temperature increases, the hydration shell of PEO is disrupted, and the hydrophobic PPO segments drive micelle aggregation. These micelles organize into a three-dimensional (3D) gel network as the critical gelation temperature is reached. PLGA, a biodegradable polymer, primarily contributes to the stability and mechanical properties of the gel matrix rather than directly influencing the thermoresponsive mechanism. The addition of PLGA microparticles enhances the structural integrity of the gel while maintaining its thixotropic and shear-thinning properties [44]. The hydrophilic nature of the PEO chains ensures compatibility with aqueous environments, while the hydrophobic interactions between PPO chains and PLGA microparticles contribute to gel network formation (synergistic effects of poloxamers and PLGA) [43]. This balance is critical for achieving the observed thermoresponsive behavior and mechanical properties.

At high shear rates, the alignment of micelles or disruption of the gel network reduces resistance to flow (shear stress). In our results, the plateau or oscillation in the blue series could be due to structural rearrangements in the gel network as the applied shear rate increases. Both gel formulations are suitable for applications requiring easy spreadability under high shear (topical drug delivery). RoEO-PLGA _A and RoEO-PLGA _B structures can recover after shear stress (RoEO-PLGA _B faster than RoEO-PLGA _A), making it ideal for applications where the material needs to hold its shape after being applied.

On the monoterpenes found in RoEO by GC–MS: α-pinene (17.4% in RoEO sample, 11.11% in RoEO Tunisia reference) is known for its strong antibacterial properties [45], particularly against *S. aureus* and *E. coli*; camphor (29.69% in RoEO sample, 9.27% in RoEO Tunisia reference) exhibits antibacterial activity, especially against Gram-positive bacteria, often used in antiseptic formulations [46]; eucalyptol (14.29% in RoEO sample, 52.77% in RoEO Tunisia reference), also known as 1,8-cineole, demonstrates broad-spectrum antibacterial effects and is effective against both Gram-positive and Gram-negative bacteria [47]; β-pinene (0.34% in RoEO sample, 5.33% in RoEO Tunisia reference) is effective against various bacterial strains and complements the activity of α-pinene [48]; borneol (4.7% in RoEO sample, 2.6% in RoEO Tunisia reference) is known for its antimicrobial properties, particularly useful in traditional medicine for wound healing [49]; terpinen-4-ol (0.59% in RoEO sample, 0.58% in RoEO Tunisia reference), commonly found in Tea Tree oil [50], demonstrates antimicrobial properties and it is effective against skin and respiratory pathogens; linalool (0.77% in RoEO sample, 0.57% in RoEO Tunisia reference) exhibits antimicrobial activity and is effective against various bacterial and fungal strains [51]; verbenone (3.19% in RoEO sample, 0.04% in RoEO Tunisia reference), often found in EOs used for respiratory issues, shows antibacterial and antifungal properties [52].

The FTIR, SEM, and DLS results together confirm the successful synthesis of PLGA microparticles encapsulating RoEO via the emulsion method. The spherical morphology and smooth surface validate proper emulsification and polymer stabilization. The elemental composition highlights the expected PLGA backbone and potentially encapsulated RoEO components.

Furthermore, the results of the DLS analysis underscore the formulation’s remarkable uniformity, highlighting the efficiency of encapsulating and evenly dispersing RoEO within the PLGA matrix. This uniformity is likely the result of meticulously optimized mixing and stabilization processes. The observed low PDI and well-controlled particle size are crucial for ensuring consistent therapeutic efficacy, as they directly influence the release profile, bioavailability, and stability of the active compounds [53].

Moreover, the micrometer-sized particles contribute to an enhanced gel texture, facilitating smooth application and promoting improved skin penetration. This combination of controlled particle size and optimized texture ensures that the formulation not only delivers the active compounds effectively but also enhances user experience, making it a promising candidate for topical therapeutic applications [53].

By combining RoEO’s potent bioactive properties with PLGA’s biocompatibility, this formulation demonstrates significant potential for drug delivery, wound healing, and cosmetic applications. The DLS analysis not only validates the quality and uniformity of the RoEO-PLGA system but also reinforces its suitability as a robust platform for advanced therapeutic and cosmetic solutions.

Compared to RoEO-PLGA _A, RoEO-PLGA _B (with 20% Poloxamer 407 and 2% Poloxamer 188, respectively) shows lower shear stress across the range of shear rates, likely due to a weaker gel network or reduced micellar density and less pronounced hysteresis, indicating faster structural recovery or less pronounced breakdown under shear.

The antimicrobial assay evaluated the effectiveness of the formulations against common bacterial and fungal strains. These strains were selected because they represent a spectrum of pathogens that the developed formulations are likely to encounter in real-world applications, such as wound care, skin infections, and fungal-related dermal conditions. Specifically, *S. aureus* is a leading cause of skin and soft tissue infections, including impetigo, folliculitis, and infected wounds [54]. It is also known for its ability to form biofilms, which complicates treatment and highlights the need for effective topical antimicrobial agents. While primarily associated with gastrointestinal infections, *E. coli* is also implicated in wound infections and other secondary skin infections, particularly in immunocompromised individuals [55]. As a common fungal pathogen, *C. albicans* causes cutaneous and mucocutaneous infections, particularly in moist or damaged skin environments [56]. Its inclusion ensures that the gel’s antifungal potential is assessed, given the prevalence of fungal co-infections in many skin conditions. This directly validated the functional performance of RoEO as an active ingredient and the gel’s potential for therapeutic use. Our RoEO-PLGA gels demonstrated significant antimicrobial activity against *S. aureus*, *E. coli*, and *C. albicans*. The IZs ranged from 9.9 to 27.2 mm, with the strongest inhibition observed against *C. albicans*. This aligns with literature findings that highlight the antimicrobial potency of major RoEO components, such as α-pinene and camphor, against Gram-positive bacteria and fungi. For instance, Borges et al. reported the antibacterial activity of α-pinene with zones of inhibition up to 20 mm against *S. aureus* [45], while Duda-Madej et al. confirmed camphor’s effectiveness against similar pathogens [46]. Fluconazole complexed with cyclodextrins has been reported to provide better antimicrobial effects than its unformulated counterpart. Our results for *S. aureus* and *E. coli* indicate moderate inhibition, which may suggest that while the formulation is effective, further optimization might be needed to match or exceed the efficacy of established commercial products [57].

These findings underscore the importance of encapsulation strategies in tailoring release profiles and optimizing antimicrobial activity for specific microbial targets. The results highlight the potential of RoEO-PLGA formulations for applications requiring sustained and targeted antimicrobial efficacy, particularly against fungal infections.

## 3. Conclusions

This study successfully demonstrated the formulation and characterization of RoEO-loaded PLGA NPs embedded in thermoresponsive poloxamer gels. Our findings include the identification of antimicrobial components in RoEO, the suitability of the gel’s pH and gelation temperature for topical applications, and favorable rheological properties such as thixotropy and shear-thinning behavior, ensuring ease of application and stability. These characteristics highlight the potential of the formulation as an effective carrier for antimicrobial therapy.

Future studies will focus on further optimizing the gel composition to enhance its performance and conducting in vivo evaluations to assess efficacy and safety in a clinical context. Additionally, advanced release studies will be performed to better understand the delivery kinetics of the active compounds, which is critical for clinical application.

## 4. Materials and Methods

### 4.1. Plant Material and Essential Oil Extraction

The plant material (leaves) of *R. officinalis* cultivated species were collected in August 2024 from southwest Romania flora (Cârcea Village, Dolj County, Oltenia Region, Romania). The vegetal samples were deposited in the Herbarium of the Department of Pharmaceutical Botany, Faculty of Pharmacy, University of Medicine and Pharmacy of Craiova. The study did not involve endangered or protected species. Samples were cleaned and naturally air-dried in shaded, cool areas, and then grounded for extraction. In a NeoClevenger-type apparatus, 100 g of died and grounded leaves were hydro-distilled for four hours with 1000 mL of distilled water, in a round-bottom flask. The mixture was heated, and the released RoEO was collected in a graded column after passing through a condenser. After hydro-distillation, extracted RoEO was collected in glass vials, dehydrated on anhydrous sodium, and stored in dark, tightly sealed bottles, at 4 °C, for further analysis.

### 4.2. Chemicals and Reagents

Poly(D,L-lactide-*co*-glycolide) acid 65:35, molecular weight (MW) 40,000–75,000, Poloxamer 407, Poloxamer 188, and RoEO (Tunis, Tunisia) reference were purchased from Sigma-Aldrich (Taufkirchen, Germany). Dichloromethane (DCM), PVA 8–88 (MW ~67,000) and purified water were purchased from Merck Millipore (Darmstadt, Germany).

### 4.3. GC–MS Analysis

This assay was performed to determine the chemical composition of RoEO. Identifying the major components was critical to understanding its potential antimicrobial properties, which underpin its role as the active ingredient in our formulations. To determine the chemical composition of RoEO, a detailed analysis was conducted using GC–MS. This method enabled the identification and quantification of individual components through comparison with reference spectra available in the National Institute of Standards and Technology (NIST) Library (2020 edition). The analytical setup utilized a Thermo Scientific Focus GC system (Norristown, PA, USA) integrated with an AI/AS 3000 autosampler and a DSQ II mass detector. A TraceGOLD TG-624 capillary column, measuring 60 m in length, 0.25 mm in internal diameter, and with a film thickness of 1.4 μm, was employed for chromatographic separation. Sample injections were performed using a volume of 1 μL, with helium serving as carrier gas at a flow rate of 1.4 mL/min. A split injection mode with a ratio of 1:50 was applied. The setup temperature of the GC oven was initiated at 90 °C, followed by an increase of 3 °C per minute until reaching 220 °C, where it was held constant for five minutes to ensure optimal separation. Mass spectrometric conditions were carefully optimized for accurate detection. The transfer line and ion source temperatures were set at 240 °C and 230 °C, respectively. Electron impact (EI) ionization was conducted at an energy level of 70 eV. Data acquisition was performed in full scan mode across a mass-to-charge (*m*/*z*) range of 50–450. Each analysis was performed in triplicate to ensure reproducibility. Retention times of the detected constituents were recorded, and their mass spectra were compared against the NIST Library Reference Database for precise identification and quantification. This comprehensive GC–MS approach facilitated a thorough profiling of RoEO, offering critical insights into its chemical composition.

### 4.4. Preparation of RoEO-PLGA Microparticles

**Figure 12:**
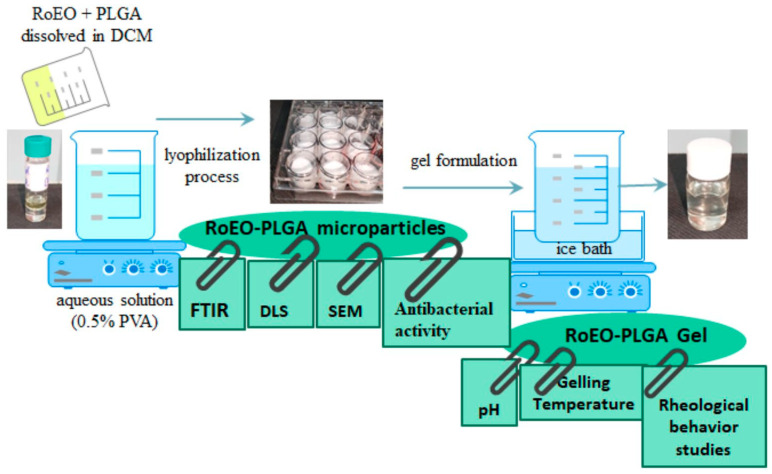
Graphic process of RoEO-PLGA microparticles and RoEO-PLGA gels formulation and characterization. DCM: Dichloromethane; DLS: Dynamic light scattering; FTIR: Fourier-transform infrared; PLGA: Poly(lactic-*co*-glycolic) acid; PVA: Poly(vinyl alcohol); RoEO: Rosemary essential oil; SEM: Scanning electron microscopy.

RoEO-PLGA microparticles were prepared by the oil–water emulsion method. Firstly, 1 mL of RoEO (extracted or Tunisia reference) was mixed with 5 mL PLGA solution (500 mg dissolved in 5 mL DCM) and stirred in a SilentCrusher vortex at 40,000 rpm. The resulting oil phase was added drop by drop in an aqueous solution (0.5% PVA) under constant stirring (900 rpm) until the entire amount of organic solvent had evaporated (five hours). The obtained particles were centrifuged (11,000 rpm; Eppendorf 5804), washed, and subjected to a lyophilization process (Alpha 1–2 LSCbasic freeze dryer, Martin Christ GmbH, Osterode am Harz, Germany), as follows: the suspended particles were frozen at −55 °C overnight and at 0.02 mbar for 48 h. RoEO-PLGA microparticles were stored in the fridge for further investigations (Figure 12).

### 4.5. Gel Formulation

In order to explore the feasibility and performance of thermoresponsive gel formulations incorporating RoEO-loaded PLGA NPs, we opted to compare the minimum and maximum concentrations of the two poloxamers (Poloxamer 407 and Poloxamer 188) as a starting point. This approach allowed us to evaluate the differences in key parameters, such as gelation temperature, pH, viscosity, and rheological behavior, and to establish a baseline understanding of their effects on the gel’s performance.

Over 100 mL aqueous suspension of RoEO-PLGA (150 mg RoEO-PLGA dispersed in cooled water to 4 °C) were added under stirring on a magnetic Raypa plate, on ice bath (A) 25% (*w*/*v*) Poloxamer 407 and 10% (*w*/*v*) Poloxamer 188 or (B) 20% (*w*/*v*) Poloxamer 407 and 2% (*w*/*v*) Poloxamer 188 (Figure 12). The resulting formulations were abbreviated as RoEO-PLGA _A and RoEO-PLGA _B, respectively.

### 4.6. FTIR Spectroscopy

FTIR data were acquired on solid state sample (directly on the lyophilized RoEO-PLGA microparticles sample to preserve the integrity of the encapsulated system, ensuring that the results reflected the true molecular composition and interactions within the formulation) using a Shimadzu AIM-9000 spectrometer, which was fitted with attenuated total reflectance (ATR) accessories (Shimadzu, Tokyo, Japan). The spectra were recorded over 20 scans, achieving a resolution of 4 cm^−1^ within the range of 4000 to 400 cm^−1^. Wavelength assignments were determined through a thorough analysis of pertinent literature.

### 4.7. XRD Analysis

XRD analysis was performed utilizing a Bruker AXS D8-Advance X-ray diffractometer (Bruker AXS GmbH, Karlsruhe, Germany), which operates with CuKα radiation (λ = 0.1541 nm). The system is equipped with a rotating sample stage and features an Anton Paar TTK low-temperature cell capable of operating between −180 °C and 450 °C, as well as a high-temperature cell that accommodates temperatures up to 1600 °C. Additionally, the apparatus maintains high vacuum conditions, an inert atmosphere, and relative humidity control. The resulting XRD patterns were systematically compared against the ICDD Powder Diffraction Database (ICDD file 04-015-9120).

### 4.8. SEM Analysis

SEM micrographs were acquired using a JSM-IT200 InTouchScope™ Scanning Electron Microscope (Freising, Germany), which is equipped with a field emission gun (FEG) and an energy-dispersive X-ray spectroscopy (EDS) system.

### 4.9. DLS Particle Size Distribution Analysis

DLS analysis was conducted using a Microtrac/Nanotrac 252 instrument (Montgomeryville, PA, USA). Each sample was measured in triplicate at RT (22 °C) and at a scattering angle of 172°.

### 4.10. Thermal Analysis

Thermal analysis was conducted to assess the thermal stability and decomposition profiles of the formulations. This was important for confirming that the encapsulated RoEO and the PLGA matrix remain stable under storage and application conditions. Thermal analysis was conducted using an aluminum crucible on TGA/DSC 3+ (Mettler-Toledo GmbH, Germany) equipped with a digital temperature and analog (DTA) sensor. The analyses were carried out under a dynamic air atmosphere, oxidative conditions with synthetic air with a flow rate of 20 mL/min, in the range of 25–400 °C, at a heating rate of 10 °C/min.

### 4.11. pH Measurement

The pH of gels was measured with a HI991003 pH-meter (Hanna Instruments, TX, USA).

### 4.12. Viscosity and Rheological Behavior Studies

Viscosity and rheological behavior studies examined the mechanical and flow properties of the gels. The rheological properties of the two formulations were analyzed using a rotational viscometer (HAAKE Viscotester 550; Thermo Scientific, Altrincham, UK) equipped with Lauda immersion thermostat A 100 and water bath 006T. Viscosity was determined at different temperatures, and at rotational speeds ranging from 3 to 600 rpm. The gelation temperature was recorded as the temperature was raised from 25 to 40 °C at a heating rate of 1 °C/min.

### 4.13. Assessment of the Antimicrobial Properties

Using the agar diffusion method, the antimicrobial activity of RoEO was further evaluated against standard bacterial strains (*S. aureus* ATCC 29213 and *E. coli* ATCC 25922) and one fungal standard strain (*C. albicans* ATCC 14053). The inoculums with 0.5 turbidity on a McFarland scale of the above-named standardized strains were applied on a Müller–Hinton agar within 15 min of preparation [58]. Blank antimicrobial susceptibility paper disks with a 6 mm diameter (Thermo Scientific, UK) were carefully infused with the test samples. These samples were carefully handled using sterilized forceps and were subsequently placed on the surface of Müller–Hinton agar plates. As a negative control, a paper disc infused with water was used. The Petri dishes were afterwards incubated at 37 °C for 24 h. The antimicrobial activity of the tested samples was established through the measurement of the total inhibition diameters of the bacterial and fungal growth, respectively, including the disk area. The diameter of inhibition zones was expressed in mm.

### 4.14. Statistical Analysis

All experiments were performed in triplicate for all samples, all calibration curves, and concentrations. Statistical analysis was carried out using Microsoft Office Excel 2019 (Microsoft Corporation, Redmond, WA, USA), and expressed as mean ± standard deviation (SD). *p*-values < 0.05 were considered statistically significant. Graphical figures were obtained with ConceptDraw Diagram software (version 18).

## Figures and Tables

**Table 1:** Compounds identified in rosemary essential oil.

No.	Compound	t_R_ (min)	RI (NIST)	RoEO	RoEO Tunisia Reference
1.	tricyclene	6.56	933	0.36	0.11
2.	camphene	7.59	950	9.76	3.27
3.	2,4-thujadiene	7.73	971	0.18	0.03
4.	β-pinene	8.73	972	0.34	5.33
5.	α-pinene	8.74	939	17.4	11.11
6.	1-octen-3-ol	8.82	1078	0.14	0.02
7.	3-octanone	9.07	1121	0.2	0.03
8.	α-myrcene	9.27	991	2.87	0.86
9.	β-thujene	9.36	964	–	0.23
10.	3-octanol	9.61	1126	–	–
11.	α-phellandrene	10.04	1015	0.16	0.11
12.	3-carene	10.14	1030	0.03	0.11
13.	α-terpinene	10.56	1016	0.63	0.36
14.	β-cymene	10.95	1018	2.02	1.57
15.	D-limonene	11.19	1031	3.59	1.86
16.	eucalyptol	11.35	1034	14.29	52.77
17.	β-*cis*-ocimene	12.12	1049	–	0.03
18.	γ-terpinene	12.72	1062	0.62	0.6
19.	linalool	15.15	1095	0.77	0.57
20.	crysanthenone	16.31	1102	0.08	–
21.	terpinolene	17.47	1088	0.38	0.26
22.	camphor	17.9	1146	29.69	9.27
23.	camphenilanol	18.31	1151	–	–
24.	sabinone	18.76	1162	0.05	–
25.	pinocarvone	18.76	1191	–	–
26.	D-pinocamphone	18.85	1206	0.09	0.02
27.	*endo*-borneol	19.4	1163	4.7	2.6
28.	terpinen-4-ol	19.95	1176	0.59	0.58
29.	α-terpinyl propionate	20.91	1333	1.58	1.78
30.	verbenone	21.56	1204	3.19	0.04
31.	*trans*-shisool	23.76	1326	–	–
32.	(+)-borneol acetate	25.45	1330	3.48	0.81
33.	(+)-*cis*-verbenol acetate	25.68	1351	–	0.01
34.	thymol	25.91	1290	0.07	–
35.	piperitenone	26.92	1303	0.05	–
36.	α-cubebene	27.61	1374	–	0.02
37.	ylangene	27.68	1395	0.06	0.05
38.	copaene	27.82	1415	0.04	0.22
39.	carvacrol	28.04	1298	0.05	–
40.	methyleugenol	28.34	1396	–	0.01
41.	caryophyllene	28.71	1420	1.47	3.77
42.	humulene	29.36	1454	–	0.38
43.	*p*-thymol	29.58	1465	–	–
44.	7-*epi*-α-cadinene	29.76	1491	0.04	0.02
45.	α-bisabolene	30.2	1506	–	0.05
46.	(-)-δ-cadinene	30.36	1520	0.11	0.23
47.	*trans*-calamenene	30.41	1542	0.02	–
48.	α-calacorene	30.72	1561	0.04	0.01
49.	(+)-sativen	30.94	1583	–	–
50.	cubenol	32.01	1600	–	–
51.	α-bisabolol	32.62	1621	0.01	0.01
52.	levomenthol	32.62	1632	0.02	–
53.	caryophyllene oxide	38.74	1652	0.02	0.1
Total No. of compounds identified				38	38
Total (%)				99.19	99.21
Monoterpene hydrocarbons (%)				38.34	25.84
Oxygenated monoterpenes (%)				58.7	68.46
Sesquiterpene hydrocarbons (%)				1.78	4.75
Oxygenated sesquiterpenes (%)				0.03	0.11
Other compounds (%)				0.34	0.05

NIST: National Institute of Standards and Technology (USA); RI: Retention index; RoEO: Rosemary essential oil; t_R_: Retention time.

**Table 2:** Gelation temperature, pH and gelation time of the two formulations (results expressed as mean ± SD).

Formulation	Gelation Temperature (°C)	pH	Gelation Time (s)
RoEO-PLGA _A	27.6 ± 0.047	6.63 ± 0.024	58
RoEO-PLGA _B	32.9 ± 0.094	6.40 ± 0.016	45

PLGA: Poly(lactic-*co*-glycolic) acid; RoEO: Rosemary essential oil; SD: Standard deviation.

**Table 3:** Comparative analysis of rheological properties of RoEO-PLGA systems.

Parameter	RoEO-PLGA _A (35 °C)	RoEO-PLGA _B (36.6 °C)	Comment
Hysteresis loop area (Pa/s)	12.02	−168.50	positive/negative value indicates a thixotropic/ rheotropic liquid
Thixotropic index (TI) (RPM1/RPM2 = 1/10)	9.48	6.86	TI > 1: shear thinning TI = 1: Newtonian liquid TI < 1: shear thickening
Energy dissipation ratio (%)	13.94	−8.34	negative value indicates the rheopectic behavior
Shear recovery (%) (shear rate s^−1^)	76.78	118.86	supra-unitary value is accounted to rheopectic behavior
Yielding stress (Pa)	0.614	14.07	indication of plastic rheology of shear thickening non- Newtonian liquid
Behavior	thixotropic, plastic non-Newtonian liquid	rheopectic, plastic non-Newtonian liquid	RoEO-PLGA_B liquid exhibits, simultaneously, antagonist behavior a plastic rheology and also rheotripsy!

PLGA: Poly(lactic-*co*-glycolic) acid; RoEO: Rosemary essential oil; RPM: Revolution per minute.

**Table 4:** Average diameters of bacterial growth inhibition zones.

Tested Microorganism	Diameter of Inhibition Area (mm)					
	1	2	3	4	5	6
*S. aureus*	14.5	14.5	12.7	12.8	0	0
*E. coli*	10.7	11.6	9.8	11.7	0	0
*C. albicans*	10.8	12.8	27.0	27.2	0	0

1: RoEO Tunisia reference; 2: RoEO; 3: RoEO-PLGA _A; 4: RoEO-PLGA _B; 5, 6: Negative controls (sterile saline solution and sterile distilled water, respectively); PLGA: Poly(lactic-*co*-glycolic) acid; RoEO: Rosemary essential oil.

## Data Availability

The original contributions presented in this study are included in the article. Further inquiries can be directed to the corresponding author.
